# Absence of Ca^2+^-Induced Mitochondrial Permeability Transition but Presence of Bongkrekate-Sensitive Nucleotide Exchange in *C. crangon* and *P. serratus*

**DOI:** 10.1371/journal.pone.0039839

**Published:** 2012-06-29

**Authors:** Csaba Konrad, Gergely Kiss, Beata Torocsik, Vera Adam-Vizi, Christos Chinopoulos

**Affiliations:** Department of Medical Biochemistry, Semmelweis University, Budapest, Hungary; Karolinska Institutet, Sweden

## Abstract

Mitochondria from the embryos of brine shrimp (*Artemia franciscana*) do not undergo Ca^2+^-induced permeability transition in the presence of a profound Ca^2+^ uptake capacity. Furthermore, this crustacean is the only organism known to exhibit bongkrekate-insensitive mitochondrial adenine nucleotide exchange, prompting the conjecture that refractoriness to bongkrekate and absence of Ca^2+^-induced permeability transition are somehow related phenomena. Here we report that mitochondria isolated from two other crustaceans, brown shrimp (*Crangon crangon*) and common prawn (*Palaemon serratus*) exhibited bongkrekate-sensitive mitochondrial adenine nucleotide transport, but lacked a Ca^2+^-induced permeability transition. Ca^2+^ uptake capacity was robust in the absence of adenine nucleotides in both crustaceans, unaffected by either bongkrekate or cyclosporin A. Transmission electron microscopy images of Ca^2+^-loaded mitochondria showed needle-like formations of electron-dense material strikingly similar to those observed in mitochondria from the hepatopancreas of blue crab (*Callinectes sapidus*) and the embryos of *Artemia franciscana*. Alignment analysis of the partial coding sequences of the adenine nucleotide translocase (ANT) expressed in *Crangon crangon* and *Palaemon serratus* versus the complete sequence expressed in *Artemia franciscana* reappraised the possibility of the 208-214 amino acid region for conferring sensitivity to bongkrekate. However, our findings suggest that the ability to undergo Ca^2+^-induced mitochondrial permeability transition and the sensitivity of adenine nucleotide translocase to bongkrekate are not necessarily related phenomena.

## Introduction

Mitochondria probed from a vast diversity of tissues and species exhibit the property of Ca^2+^ sequestration [1]; upon reaching a threshold for Ca^2+^ uptake capacity, these organelles undergo a permeability transition [2], substantiated by the assembly of pores of a sufficient size (cut-off ∼1,5 kDa). This allows the passage of solutes and water, resulting in the swelling and ultimately rupture of the outer mitochondrial membrane [3].

However, Ca^2+^ -induced permeability transition cannot be elicited in mitochondria from ghost shrimp (*Lepidophthalmus louisianensis*) [4] and embryos of brine shrimp (*Artemia franciscana*) [5]. Furthermore, mitochondria isolated from the blue crab (*Callinectes sapidus*) [6], shore crab, (also known as European green crab, *Carcinus maenas*) [7], and Caribbean spiny lobster (*Panulirus argus*) [8], also belonging to the crustacean subphylum exhibited impressive calcium uptake capacities. While lack of PTP has not been explicitly sought, mitochondria from the latter organisms did not display spontaneous losses of sequestered calcium. On the other hand, cadmium, which is known to induce permeability transition [9], causes *in situ* mitochondrial swelling in freshwater crab (*Sinopotamon yangtsekiense*) [10], and the crustacean isopod *Jaera nordmanni* [11]. This allows the suggestion that crustaceans lack the specific machinery for orchestrating the Ca^2+^-induced permeability transition pore (PTP).

Despite intense research since the characterization of PTP by Hunter and Haworth in 1979 [12]–[14], the identity of the proteins comprising it remain unknown, reviewed in [15], [16]. Among candidate proteins, cyclophilin D and the adenine nucleotide translocase (ANT) has gained support as playing a modulatory rather than structural role in pore formation [17]–[23]. This has been firmly established by extensive literature on the effects of ANT ligands and cyclophilin D inhibitors on mitochondrial Ca^2+^ uptake capacity, reviewed in [24]–[26], see also [27] and [28].

Mindful of i) the well-established ligand-profile of the ANT, ii) the modulatory role of ANT in mammalian PTP, and iii) the absence of Ca^2+^-induced PTP in mitochondria from the embryos of *Artemia franciscana*, we recently addressed the effect of ANT ligands on Ca^2+^ uptake capacity in mitochondria isolated from brine shrimp embryos. We found that in mitochondria of *Artemia franciscana* the ADP-ATP exchange rate mediated by the ANT and Ca^2+^ uptake capacity were insensitive to bongkrekic acid [29]. We examined the possibility that refractoriness to this poison may be a direct consequence of the amino acid sequence of the *Artemia* ANT. Indeed, sequencing of the *Artemia* mRNA coding for ANT revealed that it transcribed a protein with a stretch of amino acids in the 198-225 region –within a broader highly conserved region- exhibiting only a 48–56% similarity to those from other species, including the deletion of three amino acids in positions 211, 212 and 219 [29].

Based on our findings with the *Artemia franciscana* mitochondria it was postulated that the sensitivity of the ANT to BKA and the Ca^2+^-induced permeability transition are somehow related. To scrutinize this claim, mitochondria isolated from related species were probed. We hereby show that mitochondria from the malacostraca *Crangon crangon* and *Palaemon serratus* lacked a Ca^2+^-induced PTP, yet ADP-ATP exchange mediated by the ANT was sensitive to BKA. In addition, the ANT carriers of *Crangon crangon* and *Palaemon serratus* exhibited different amino acid sequences in the 208-214 region compared to that expressed in *Artemia franciscana*.

## Results

### *Crangon Crangon* and *Palaemon serratus* Mitochondria Lack the Ca^2+^-induced Permeability Transition Pore

**Figure 1:**
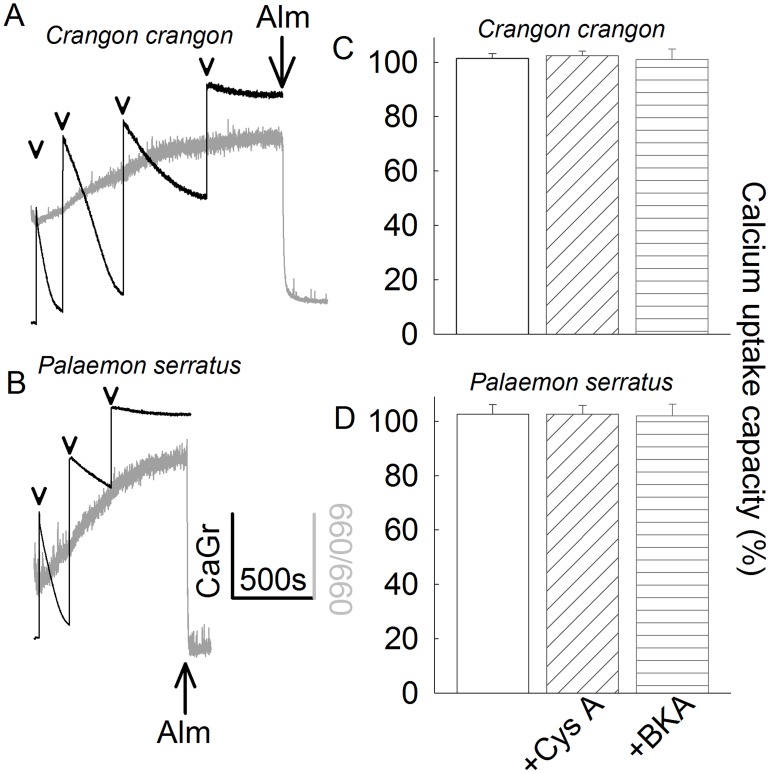
Characterization of Ca^2+^ retention of *Crangon crangon* (A, C) and *Palaemon serratus* (B, D) mitochondria. A, B : Time courses of CaGr-5N fluorescence (arbitrary units, black traces) reflecting extramitochondrial [Ca^2+^], in parallel to light scattering at 660/660 nm (arbitrary units, grey traces), reflecting mitochondrial swelling. 200 µM CaCl_2_ was added where indicated by the arrowheads. 40 µg of alamethicin (Alm) was added where indicated. Results shown in panels A and B are representative of at least 4 independent experiments. **C, D**: Bar graphs of maximum Ca^2+^ uptake capacity (% scale) calculated from the number of 100 µM CaCl_2_ additions given to mitochondria until no further decrease in CaGr-5N fluorescence (implying maximum mitochondrial calcium uptake) was observed, in the presence of 1 µM cyclosporin A or 20 µM BKA, (n = 3). The rates of Ca^2+^ uptake of the last addition were compared among treatment groups, where it exhibited a small variability, but no statistical significance (p = 0.933 for *Crangon crangon* and p = 0.989 for *Palaemon serratus*, ANOVA on Ranks). The rates of Ca^2+^ uptake of all previous CaCl_2_ additions were virtually identical among all treatment groups.

Mitochondria were challenged to sequester several boluses of 200 µM CaCl_2_ (arrowheads in figures 1A and 1B), while recording Calcium Green 5N 6K^+^ salt fluorescence, reflecting extramitochondrial [Ca^2+^] (black traces). In parallel measurements, light scatter at 660 nm was recorded as an indication of mitochondrial swelling (grey traces). Towards the end of the light scatter recordings, 40 µg of the pore-forming peptide alamethicin was added (Alm; where indicated) causing maximal swelling, as a calibration standard. Mitochondria were able to sequester large amounts of calcium but showed no evidence of swelling long after reaching their maximum Ca^2+^ uptake capacity. Mitochondria were releasing Ca^2+^ to the medium promptly upon addition of an uncoupler (not shown). These experiments were performed in the absence of exogenously added adenine nucleotides, which are known to increase maximum Ca^2+^ uptake capacity in mammalian mitochondria [30]. Mitochondria obtained from *Crangon crangon* were able to accumulate approximately 1.2 µmol of Ca^2+^ per mg protein, while those obtained from *Palaemon serratus* were able to accumulate approximately 0.6 µmol of Ca^2+^ per mg protein, at which point they were starting to sediment in the cuvette. This precluded the possibility for obtaining fluorimetric recordings for prolonged periods of time. The extent of contamination (as deduced from electron microscopy images) from non-mitochondrial material in these preparations amounted to more than 40%. Therefore, the true maximum Ca^2+^ uptake capacity per mg mitochondrial protein is even higher. Nonetheless, these values were unaffected by the presence of either cyclosporin A (cys A) or bongkrekic acid, as shown in panels 1C and 1D. This is in accordance with the assumption that without PTP, neither cys A nor BKA has an effect on Ca^2+^ uptake.

**Figure 2:**
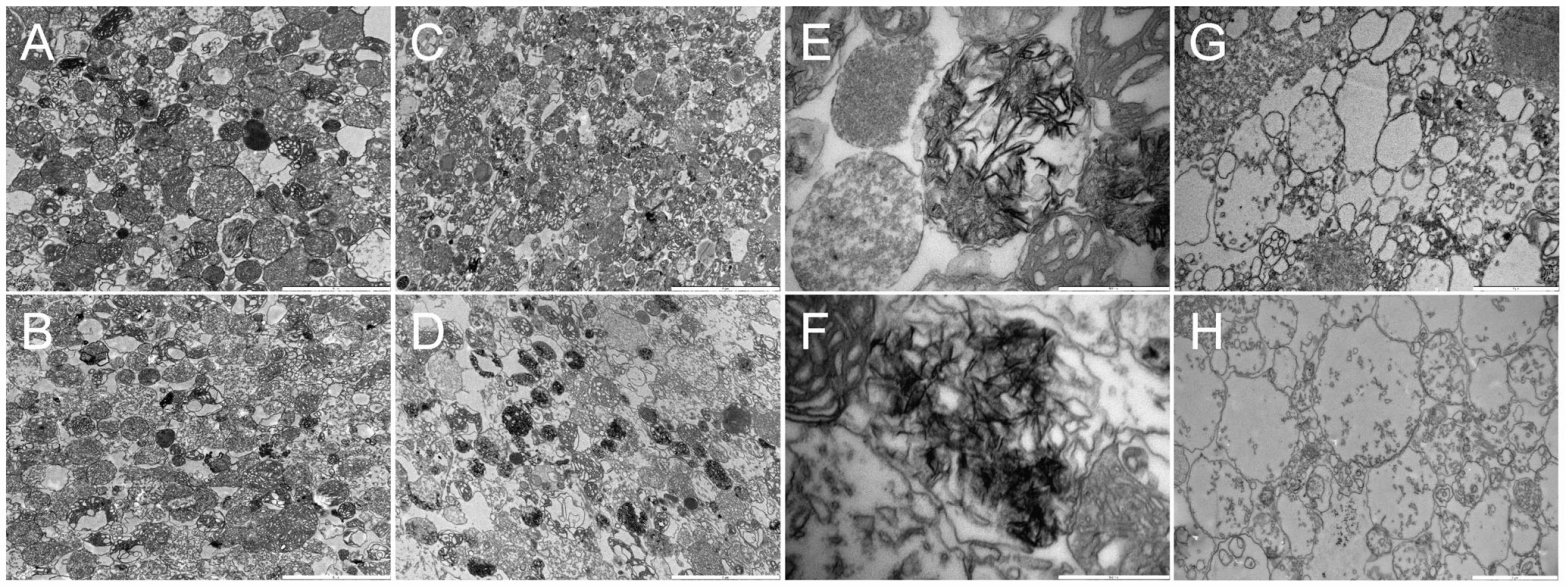
TEM images of *Crangon crangon* (A, C, E, G) and *Palaemon serratus* (B, D, F, H) mitochondria. A, B : Mitochondria were fixed after incubating in the absence of Ca^2+^ for 1 hour. **C, D**: Mitochondria were treated as in experiments shown in panels 1A and 1B and fixed 2 hours after the last addition of 200 µM CaCl_2_. **E, F**: as in **C, D**, but viewed at a higher magnification, demonstrating the needle-like appearance of calcium phosphate mitochondrial precipitates**.** **G, H**: Mitochondria treated with 40 µg of alamethicin. Bars shown in the lower right corners of each panel are as follows: A, B C, D: 5 µm, E, F: 500 nm, G, H: 2 µm.

To verify this assumption, mitochondria were fixed for viewing using transmission electron microscopy. In figure 2, panels A, C, E, and G depict *Crangon crangon* mitochondria, and panels B, D, F and H represent those obtained from *Palaemon serratus*. Panels A and B depict mitochondria fixed after incubating in the absence of Ca^2+^ for 1 hour. In panels C and D, mitochondria were treated as in experiments shown in figure 1 and fixed 2 hours after the last addition of 200 µM CaCl_2_. Panels E and F show mitochondria treated as described for panels C and D but viewed at a higher magnification, demonstrating the needle-like appearance of calcium phosphate precipitates. This was also observed in Ca^2+^ loaded mitochondria from blue crabs [6], [31] and *Artemia franciscana* [29], in stark contrast to the ring-like structures observed in Ca^2+^-loaded mammalian mitochondria [32]. The significance of this observation –if any- is unknown, other than it appears to be a general phenomenon among crustaceans, possibly underlying a common precipitation mechanism. Panels G and H depict mitochondria treated with 40 µg of alamethicin, demonstrating that under these experimental conditions the organelles were capable of swelling. The conclusions drawn from these images are that mitochondria from *Crangon crangon* and *Palaemon serratus* i) form calcium-phosphate precipitates similar to those observed in *Artemia* cysts mitochondria, ii) do not exhibit Ca^2+^-induced swelling and iii) are able to swell upon addition of a pore-forming peptide. Collectively, from the results obtained from figures 1 and 2, we conclude that mitochondria from *Crangon crangon* and *Palaemon serratus* do not exhibit the Ca^2+^-induced permeability transition. It is also notable that in Ca^2+^-loaded mitochondria from *Crangon crangon* and *Palaemon serratus* pretreated with oligomycin there was no swelling observed upon addition of an uncoupler in the presence of phosphate carrier blockers (not shown); this scheme caused an immediate and precipitous opening of the PTP in mammalian mitochondria [33].

### ADP-ATP Exchange in *Crangon Crangon* and *Palaemon serratus* mitochondria is sensitive to BKA

Similarly to *Artemia* mitochondria, BKA exhibited no effect in those isolated from *Crangon crangon* and *Palaemon serratus*, on Ca^2+^ uptake capacity. In [29], we showed that the ADP-ATP exchange mediated by the ANT of *Artemia franciscana* is refractory to this poison. We therefore questioned if ADP-ATP exchange in *Crangon crangon* and *Palaemon serratus* mitochondria is also insensitive to BKA. The effect of BKA was tested on i) ADP-induced depolarization while recording safranine O fluorescence, and ii) on ADP-ATP exchange rate deduced from measurements of extramitochondrial [Mg^2+^], as detailed in [34]. The effect of BKA on mitochondrial respiration could not be reliably tested because of a very high rate of oxygen consumption in the absence of exogenously added adenine nucleotides, see panel 3E. This was not due to a high fraction of ‘leaky’ mitochondria, because safranine O fluorescence measurements reflecting ΔΨm prompt ADP-induced depolarizations (see below) implying the presence of sufficiently polarized, thus intact mitochondria. A respiratory control ratio for both *Palaemon serratus* (panel 3E) and *Crangon crangon* (not shown) mitochondria was ∼2. Oxygen consumption was entirely cyanide-sensitive. The addition of dithionite after cyanide showing the disappearance of the remaining oxygen in the buffer implied that the effect of cyanide was genuine, and did not coincide with the depletion of oxygen from the medium. Uncoupled respiration (induced by SF 6847) was significantly higher than state 3 respiration. This is in line with the above claim that these mitochondrial preparations did not have a high fraction of ‘leaky’ mitochondria.

**Figure 3:**
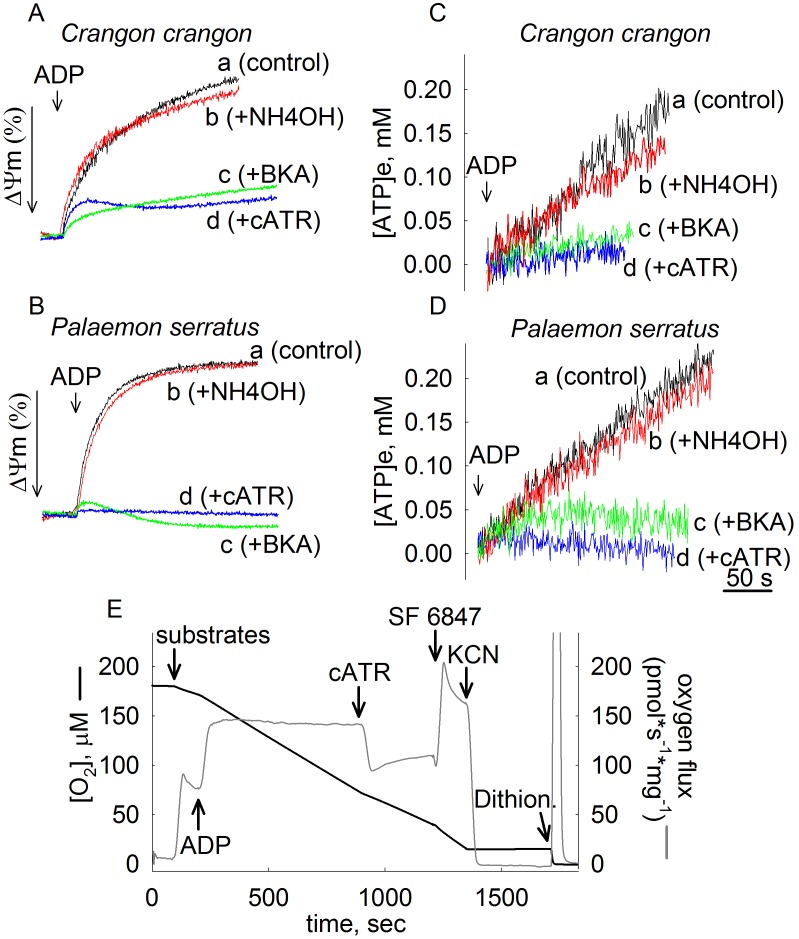
Effect of ANT ligands on ADP-induced alterations in ΔΨm and ADP-ATP exchange rates in *Crangon crangon* (A, C) and *Palaemon serratus* (B, D) mitochondria. **A, B**: Time courses of safranine O fluorescence reflecting ΔΨm (% scale). Maximum (100%) polarization is the value of safranine O fluorescence prior to the addition of ADP; minimum (0%) polarization is the value of safranine O fluorescence after the addition of 1 µM SF6947 (omitted from the graph). The length of the arrow representing ΔΨm in the y-axis is equal to a 10% change. **C, D**: Reconstructed time courses of extramitochondrial [ATP] appearing in the medium upon addition of ADP (where indicated), and effect of mitochondrial inhibitors. Vehicle of BKA (2 mM NH_4_OH, traces b), bongkrekic acid (BKA, 20 µM, traces c), or carboxyatractyloside (cATR, 1 µM, traces d) were present prior to the addition of ADP. In trace a no inhibitor was present prior to the addition of ADP. **E**: **Characterization of oxygen consumption of** ***Palaemon serratus*** **mitochondria.** Black trace represents a time course of oxygen consumption, by 0. 5 mg *Palaemon serratus* mitochondria suspended in a 2 ml volume. Grey trace represents the negative time derivative of oxygen concentration, divided by mitochondrial mass per volume. Additions of substances are indicated by arrows. ADP: 0.2 mM. cATR: 2 µM. SF 6847: 1 µM. KCN: 1 mM. Dithion.: Dithionite (added in excess). Substrates were glutamate and malate and succinate (5, 5, and 5 mM). Results shown in all panels are representative of at least 4 independent experiments.

Safranine O fluorescence recordings reflecting ΔΨm (in percentage scale) are shown in figure 3, panels A and B for *Crangon crangon* and *Palaemon serratus* mitochondria, respectively. As shown in panels 3A and 3B, addition of 2 mM ADP to mitochondria induced a decrease in ΔΨm (traces a). In the presence of 20 µM BKA (traces c) or 1 µM carboxyatractyloside (cATR, traces d), ADP-induced depolarization was largely dampened. NH_4_OH (traces b) is the solvent of BKA, and in agreement with our earlier findings [29], [34] it has a minor effect on its own due to matrix alkalinization. Likewise, recordings of extramitochondrial [Mg^2+^] converted to [ATP] appearing in the medium, indicated that ADP-ATP exchange rates were largely abolished by BKA and cATR (panels 3C and 3D for *Crangon crangon* and *Palaemon serratus* mitochondria, respectively). It is notable though that the rate of ADP-ATP exchange in *Crangon crangon* and *Palaemon serratus* mitochondria is 4-5 times slower than that from *Artemia* mitochondria (even accounting for the extent of contamination of our mitochondrial preparations by over 40%, see above). ADP-ATP exchange rate in isolated mitochondria mediated by the ANT depends on no less than 30 parameters [35], and it is beyond the scope of the present study to elaborate on the possible explanations for this phenomenon. Nevertheless, from the results shown in figure 3 it could be concluded that the ANTs expressed in *Crangon crangon* and *Palaemon serratus* are sensitive to BKA.

**Figure 4:**
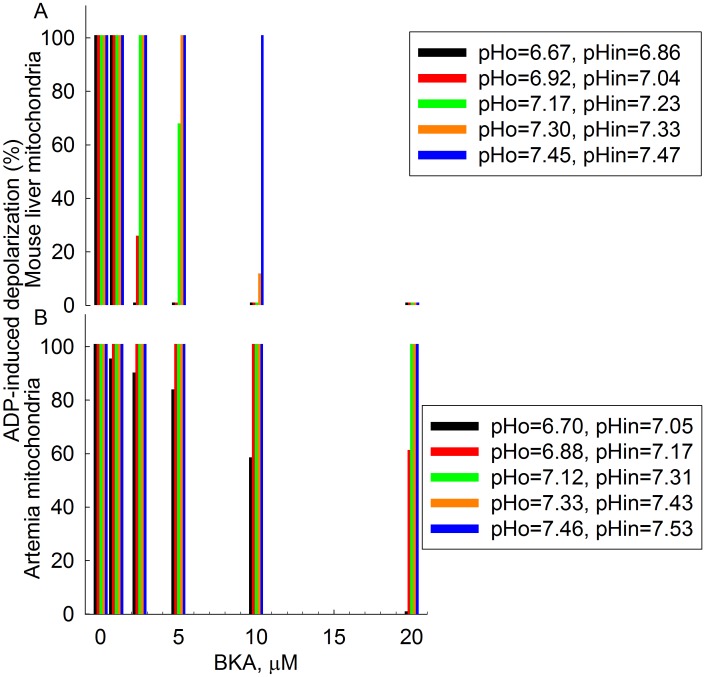
ADP-induced depolarization (% values) in mouse liver (A) and *Artemia* cyst (B) mitochondria subjected to various pH_o_, as a function of BKA concentration (0, 1.25, 2.5, 5, 10 and 20 µM). In the insets to the right the values of pH_in_ as a function of pH_o_ is shown. Data represent a representative experiment (from three independent experiments) performed in a single run. Experimental data were not pooled in order to be presented as a bar graph with SE bars because pH_o_ showed a slight variation among different experiments.

This finding prompted us to scrutinize further our previous report on *Artemia franciscana*, an organism that is phylogenetically very close to *Crangon crangon* and *Palaemon serratus* but exhibits BKA-insensitive mitochondria. The effect of BKA is dependent on pH; BKA requires to become protonated in order to exert its action [36], and as such it becomes less effective at increasing pH. It is therefore possible, that the matrix of *Artemia* mitochondria is sufficiently alkaline in order to prevent the sufficient protonation of BKA and thus its mode of action inhibiting the ANT. To test this hypothesis we performed a series of experiments recording the extent of ADP-induced depolarization (measured by safranine O) in a range of BKA concentrations (0, 1.25, 2.5, 5, 10 and 20 µM) for a range of buffers with pH_o_ varying from 6.67 to 7.46 for both *Artemia* cyst and mouse liver mitochondria. Mouse liver was used as a tissue known to exhibit sensitivity to BKA where we could therefore establish the pH range at which BKA becomes ineffective. A complete series of such an experiment is shown in figure 4. ADP-induced depolarization (% value) as a function of BKA concentration for various pH_o_ indicated in the insets on the right is shown for mouse liver mitochondria (panel A) and *Artemia* mitochondria (panel B). In these settings, the more effective BKA is, the smaller the ADP-induced depolarization. It is evident that at pH_o_ = 7.3, (i.e. pH_in_ = 7.33, orange bar panel A), 10 µM BKA was sufficient to almost completely block ADP-induced depolarization, i.e. inhibit the ANT in mouse liver mitochondria. At almost the same pH_in_, in *Artemia* mitochondria not even double the amount of BKA could affect ADP-induced depolarization (green bar, panel B). In *Artemia* mitochondria only at pH_o_ = 6.7 (i.e. pH_in_ = 7.05) was 20 µM BKA effective, a pH value at which there must be very substantial BKA protonation. At pH_o_ 7.25 in *Crangon crangon* and *Palaemon serratus* mitochondria pH_in_ should be no less than 7.35; however, they were still BKA-sensitive. From these experiments we reaffirm the refractoriness of *Artemia* mitochondria to BKA.

### Multiple Alignment Analysis of the Partial Sequences of the ANTs Expressed in *Crangon crangon, Palaemon serratus* and Other Organisms and Positioning in the Phylogenetic Tree

**Figure 5:**
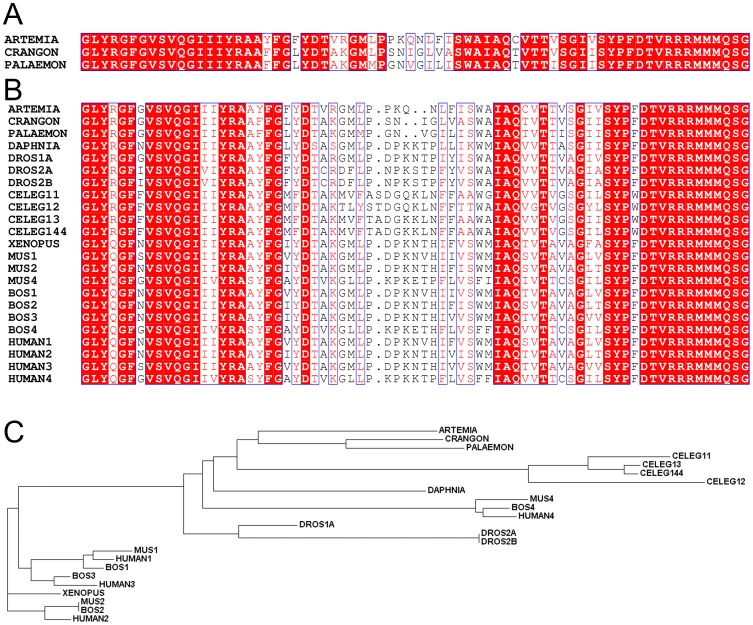
Multiple sequence analysis of the ANT expressed in various organisms and their position in the phylogenetic tree. A: Alignment of predicted primary amino acid sequences (in single-letter code) of ANT (partial sequence) expressed in *Artemia* cysts, *Crangon crangon* and *Palaemon serratus*. **B**: Alignment of predicted primary amino acid sequences expressed in various organisms (partial sequences). DAPHNIA: *Daphnia pullex*; DROS: *Drosophila melanogaster*; CELEG: *C. elegans*; XENOPUS: *Xenopus laevis*; MUS: *Mus musculus*; BOS: *Bos Taurus*. Numbers after each abbreviation indicates isoform. ‘11’, ‘12’, ‘13’ and ‘144’ indicate isoforms 1.1, 1.2, 1.3 and 1.44, respectively. Conserved regions are highlighted in red. **C**: Cladogram based on the sequence alignment shown in panel B for the same organisms.

The results obtained above prompted us to clone and sequence the ANT of *Crangon crangon* and *Palaemon serratus*. PCR products were sequenced and the final assembled nucleotide sequences were submitted to GenBank (accession numbers: JQ269837 and JQ269838 for *Palaemon serratus* and *Crangon crangon*, respectively). Alignments of the predicted ANT sequences are shown in figure 5, panels A and B. In panel A, the partial sequences expressed in *Crangon crangon* and *Palaemon serratus* are compared to that expressed in *Artemia franciscana* (accession number: HQ228154). In panel B, several more organisms are added to the sequence alignment. The inclusion of other organisms to the alignment changes the numbering of the amino acid sequences. This is partially because they are distally related, as demonstrated in the cladogram shown in panel C. *Crangon crangon, Palaemon serratus* and *Artemia franciscana* cluster in a phylogenetically close region, compared to the rest of the organisms used in the multiple sequence alignment shown in panel B. To the best of our knowledge, the sequences of the ANTs expressed in organisms belonging to the same phylogenetic branch (notably malacostraca, upper cluster of panel C) but other than *Artemia*, *Crangon* and *Palaemon*, are not known. Nonetheless, within a highly conserved region of amino acid sequence from position 176 to 245, the stretch 208-214 (PKQNLFI sequence from panel A; this numbering does not apply for the alignment shown in panel B) exhibits a low degree of homology among the BKA-sensitive crustacea *Crangon crangon* and *Palaemon serratus* and the BKA-insensitive *Artemia franciscana*. It cannot be overemphasized that there can still be additional ANT isoforms in *Crangon crangon* and *Palaemon serratus*, since we used their abdominal muscles as a whole, and not an individual organ was dissected (for the purpose of bulk generation for obtaining sufficient yields for mitochondrial isolation). However, an exhaustive search of different primers based on multiple sequence alignment between *Artemia franciscana*, *Branchiostoma floridae*, *Caenorhabditis elegans* and *Drosophila melanogaster* homologues of ANT, yielded only one suitable transcript that was sufficiently long and contained the signature sequence RRRMMM [37], [38].

## Discussion

In a previous study [29], we reported that mitochondria isolated from the cysts of *Artemia franciscana* exhibited BKA-insensitive adenine nucleotide exchange. This finding, in conjunction to the report by Menze et al. [5] showing that these mitochondria did not exhibit the Ca^2+^-induced PTP in the presence of a profound Ca^2+^ uptake capacity, prompted us to postulate that refractoriness of ADP/ATP exchange to BKA and lack of PTP are somehow related phenomena. To scrutinize this claim, we probed mitochondria isolated from the related species *Crangon crangon* and *Palaemon serratus* for sensitivity of ADP/ATP exchange to BKA and Ca^2+^-induced mitochondrial swelling. The key findings of the present study are that mitochondria isolated from the two malacostraca lacked a Ca^2+^-induced PTP and displayed the same pattern of calcium-phosphate precipitation in their matrix as in *Artemia franciscana*, yet ADP-ATP exchange mediated by the ANT was sensitive to BKA. In addition, the ANT carriers of *Crangon crangon* and *Palaemon serratus* exhibited different amino acid sequences in the 208-214 region, compared to that expressed in *Artemia* cysts. The latter finding may assist in the identification of the binding site of BKA on ANT, which is still unknown [26].

In view of a large body of work on crustacean mitochondria [4]–[8] and this one, the statement that Ca^2+^-induced PTP is absent in this subphylum starts to consolidate. Notably, Ca^2+^-induced cyclosporin A-insensitive PTP was documented in yeasts [39], organisms that belong to more ancient taxa than the crustaceans in the phylogenetic tree, prompting the conjecture that PTP as a trait was lost upon branching of the crustacean subphylum. Future revelations on PTP-lacking crustaceans and sequencing of their ANTs may lead to identification of pore modulating regions and/or the BKA binding site.

## Materials and Methods

### Ethics Statement

All procedures involving mice were carried out according to the local animal care and use committee (Egyetemi Allatkiserleti Bizottsag) guidelines.

### Mitochondrial Isolation

*Crangon crangon* and *Palaemon serratus* were obtained from Service d’Expédition de Modèles Biologiques - CNRS/FR2424 - Station Biologique de Roscoff, France. Animals were kept in aquariums filled with artificial sea water and algae at 6–8°C on 12 hours light/dark illumination cycles, until use. No ethical permissions are required for handling invertebrates for our research purposes. 10-15 animals were pooled for each preparation. The cephalothorax of each animal was removed and then the abdominal muscle was de-shelled, chopped and homogenized in ice-cold buffer containing, in mM: mannitol 225, sucrose 125, Hepes 5, EGTA 1, and 1 mg ml^−1^ bovine serum albumin (BSA, fatty acid-free), with the pH adjusted to 7.4 using Trizma. The homogenates were passed through one layer of muslin and centrifuged at 1,250 g for 10 min; the pellets were discarded, and the supernatants were centrifuged at 10,000 g for 10 min; this step was repeated once. At the end of the second centrifugation, the supernatants were discarded, and the pellets were suspended in 0.15 ml of the same buffer with 0.1 mM EGTA. Mitochondrial isolation from rehydrated *Artemia* cysts was performed as described in [29]. Mitochondrial isolation from C57Bl/6N mouse liver was performed as described in [40] with minor modifications detailed in [22]. The mitochondrial protein concentration was determined using the bicinchoninic acid assay [41].

### [Mg^2+^]_f_ Determination from Magnesium Green Fluorescence in the Extramitochondrial Volume of Isolated Mitochondria and Conversion to ADP-ATP Exchange Rate

Mitochondria (1 mg) were added to 2 ml of an incubation medium containing, in mM: mannitol 225, sucrose 125, Hepes 5, EGTA 0.1, KH_2_PO_4_ 10, MgCl_2_ 1, glutamate 5, malate 5, succinate 5, 0.5 mg/ml bovine serum albumin (fatty acid-free), pH = 7.25 (KOH), supplemented with 50 µM A_p_5A, and 2 µM Magnesium Green 5K^+^ salt. Magnesium Green (MgG) fluorescence was recorded in a Hitachi F-4500 spectrofluorimeter (Hitachi High Technologies, Maidenhead, UK) at 5 Hz acquisition rate, using 506 and 531 nm excitation and emission wavelengths, respectively. Experiments were performed at 27°C. At the end of each experiment, minimum fluorescence was measured after addition of 5 mM EDTA, followed by the recording of maximum fluorescence elicited by addition of 25 mM MgCl_2_. The affinity constants of ADP and ATP for Mg^2+^ were determined as described in [34] for the specific buffer composition and temperature and estimated as Kd_ATP_ = 0.04+/−0.003 mM, and Kd_ADP_ = 0.35+/−0.001 mM. The affinity constant of MgG for Mg^2+^ was determined as described in the supplementary material of reference [42] for the specific buffer composition and temperature and was estimated as 1.0 mM. Extrapolation to free Mg^2+^ concentration and conversion to rate of ATP appearing in the medium following addition of ADP to energized mitochondria was performed as detailed in [34].

### Mitochondrial Respiration

Oxygen consumption was performed polarographically using an Oxygraph-2k (Oroboros Instruments, Innsbruck, Austria). 0.5 mg of mitochondria were suspended in 2 ml incubation medium, the composition of which was identical to that for [Mg^2+^]_f_ determination. Experiments were performed at 27°C. Oxygen concentration and oxygen flux (pmol·s^−1^·mg^−1^; negative time derivative of oxygen concentration, divided by mitochondrial mass per volume) were recorded using DatLab software (Oroboros Instruments).

### [Ca^2+^]o Determination by Calcium Green 5N Fluorescence

Extramitochondrial [Ca^2+^] was estimated fluorimetrically with the membrane-impermeable calcium green 5N hexapotassium salt (CaGr-5N) [43], using buffer composition and conditions identical to those for [Mg^2+^]_f_ determination, but including 0.5 µM CaGr-5N instead of MgG, and using 505 and 535 nm excitation and emission wavelengths, respectively. Experiments were performed at 27°C. The physiological temperature for *Crangon crangon* and *Palaemon serratus* is between 6–25°C. We performed all experiments at 27°C because –at least for mammalian mitochondria- Ca^2+^ uptake slows down sharply below 22–24°C.

### Membrane Potential (ΔΨm) Determination in Isolated Mitochondria from *Crangon crangon* and *Palaemon serratus*

ΔΨm was estimated fluorimetrically with safranine O [44], using buffer composition and conditions identical to those for [Mg^2+^]_f_ determination, but including 5 µM safranine O instead of MgG, and using 495 and 585 nm excitation and emission wavelengths, respectively. ΔΨm was expressed in a percentage scale, assuming that addition of substrates to mitochondria causes 100% polarization, and that addition of 1 µM of the uncoupler SF 6847 causes a complete collapse of membrane potential (0% polarization). Experiments were performed at 27°C. For mouse liver and *Artemia* cyst mitochondria, buffer compositions and other experimental parameters were as detailed in [22] and [29], respectively.

### Mitochondrial Swelling

Swelling of isolated mitochondria was assessed by measuring light scatter at 660 nm using buffer composition and conditions identical to those for [Mg^2+^]_f_ determination, but in the absence of a fluorescent dye. At the end of each experiment, the non-selective pore-forming peptide alamethicin (40 µg) was added as a calibration standard causing maximal swelling. Experiments were performed at 27°C.

### Mitochondrial Matrix pH (pHi) Determination of Mouse Liver and Artemia Cyst Mitochondria

The pH_in_ of mouse liver and *Artemia* cyst mitochondria mice was estimated as described previously [45], with minor modifications. Briefly, mouse liver mitochondria (20 mg) were suspended in 2 ml of medium containing (in mm): 225 mannitol, 75 sucrose, 5 Hepes, and 0.1 EGTA [pH 7.4 using Trizma, Sigma (St Louis, MO, USA)] and incubated with 50 µm BCECF-AM (Invitrogen, Carlsbad, CA, USA) at 30°C. After 20 min, mitochondria were centrifuged at 10,600 *g* for 3 min (at 4°C), washed once and re-centrifuged. The final pellet was suspended in 0.2 ml of the same medium and kept on ice until further manipulation. A similar procedure was used for *Artemia* cysts with the exception that the medium consisted of 500 mM sucrose, 150 mm KCl, 1 mm EGTA, 0.5% (w/v) fatty acid-free BSA, and 20 mm K^+^-Hepes (pH 7.5), and the temperature was 27°C. Fluorescence of hydrolyzed BCECF trapped in the matrix was measured in a Hitachi F-4500 spectrofluorimeter in a ratiometric mode at a 2 Hz acquisition rate, using excitation and emission wavelengths of 450/490 nm and 531 nm, respectively. Buffer composition for mouse liver mitochondria was, in mM: KCl 8, K-gluconate 110, NaCl 10, Hepes 10, KH_2_PO_4_ 10, EGTA 0.005, mannitol 10, MgCl_2_ 1, glutamate 5, malate 5, 0.5 mg·ml^-1^ BSA (fatty acid-free), pH 7.25, and 50 µM Ap5A. For *Artemia* cysts, buffer composition was: 500 mm sucrose, 150 mm KCl, 20 mm Hepes (acid), 10 mm KH_2_PO_4_, 5 mm glutamate, 5 mm malate, 5 mm succinate, 1 mm MgCl_2_, 5 mg·mL^−1^ BSA (fatty-acid free), pH 7.5. The BCECF signal was calibrated using a range of buffers of known pH in the range 6.6–7.6, and by equilibrating matrix pH to that of the experimental volume by 250 nm SF 6847 plus 1 µm nigericin. For converting BCECF fluorescence ratio to pH, we fitted the function: *f*  =  *a* × exp[*b*/(*x* + *c*)] to BCECF fluorescence ratio values, where *x* is the BCECF fluorescence ratio, *a*, *b* and *c* are constants and *f* represents the calculated pH. The fitting of the above function to BCECF fluorescence ratio values obtained by subjecting mitochondria to buffers of known pH returned *r* ^2^>0.99 and the SE of the estimates of *a* and *c* constants were in the range 0.07–0.01, and <0.1 for *b*.

### Transmission Electron Microscopy (TEM)

TEM was performed exactly as described previously for mitochondria isolated from *Artemia franciscana* [29].

### Partial Sequencing of mRNA Transcribing ANT

∼1 gr of de-shelled abdominal muscle (devoid of the cephalothorax) of *Crangon crangon* and *Palaemon serratus* were each homogenized in ice-cold TRIzol Reagent (Invitrogen, Carlsbad, CA, USA) using a glass-Teflon homogenizer. Total RNA isolated by TRIzol, was reverse transcribed using Superscript II (Invitrogen) according to the manufacturer’s instructions. The cDNA obtained was used as a template for amplification by PCR reaction. Primers used were: For *Crangon crangon*, forward: GTTGACAAGAAGACCCAATTCTGG; reverse: CCAGACTGCATCATCATTCG. For *Palaemon serratus*, forward: AGTGGACAAGAAGACCCAGTTCTGG; reverse: CCAGACTGCATCATCATGCG. PCR products were cloned into the pCR®2.1-TOPO® vector and transformed into E. coli TOP10 (TOPO TA Cloning Kit, Invitrogen). Sequencing of the colonies was performed by AGOWA GmbH, Berlin, Germany.

### Multiple Sequence Alignment

Multiple sequence alignment was performed by Clustal Omega [46], and the output was generated by ESPript [47]. Cladogram was assembled by Clustal W2 [48].

### Reagents

Standard laboratory chemicals, cyclosporin A, Durcupan, gluteraldehyde, uranyl acetate, lead citrate, P1,P5-Di(adenosine-5′) pentaphosphate (A_p_5A), and safranine O were from Sigma (St. Louis, MO, USA). SF 6847 was from Biomol (BIOMOL GmbH, Hamburg, Germany). Magnesium Green 5K^+^ salt and Calcium Green 5N 6K^+^ salt were from Invitrogen (Carlsbad, CA, USA). Bongkrekic acid was from Merck (Merck KGaA, Darmstadt, Germany). All mitochondrial substrate stock solutions were dissolved in bi-distilled water and titrated to pH = 7.0 with KOH. ADP was purchased as a K^+^ salt of the highest purity available (Merck) and titrated to pH = 6.9 with KOH. Bongkrekic acid was dissolved in 1 M ultrapure NH_4_OH in 10 mM stocks and kept at −20°C.

### Statistics

Data are presented as ± standard error of the mean; significant differences between two sets of data were evaluated by t-test analysis, with P<0.05 considered to be significant, and if there were more than two groups of data, ANOVA on Ranks analysis was performed, with P<0.05 considered to be significant. Wherever single graphs are presented, they are representative of at least three independent experiments.
